# Ultrasound in rheumatoid arthritis - volar versus dorsal synovitis evaluation and scoring

**DOI:** 10.1186/1471-2474-12-124

**Published:** 2011-06-03

**Authors:** V Vlad, F Berghea, S Libianu, A Balanescu, V Bojinca, C Constantinescu, M Abobului, D Predeteanu, R Ionescu

**Affiliations:** 1 Research Center for Rheumatic Diseases, Sf. Maria University Hospital, Bucharest, Romania

**Keywords:** Ultrasonography, Rheumatoid Arthritis, synovitis, volar

## Abstract

**Background:**

Assessment of synovitis in Rheumatoid Arthritis (RA) is a major issue for a proper treatment administration; it has been proven that ultrasound (US) examination could be of valuable help and it is currently being investigated as a possible outcome measure for the disease. It is, though, of greatest importance to accurately establish the place of US scores among the already validated outcome measures, according to Outcome Measures for Rheumatoid Arthritis in Clinical Trials (OMERACT) filter. The present study is designed to compare the results of gray-scale ultrasound (GSUS) and Power Doppler ultrasound (PDUS) additive scores, separately calculated for volar and dorsal aspects of the hand, with physical examination, patient's evaluation of disease pain and global activity on Visual Analogic Scale (VAS) and traditional scores for disease activity assessment (DAS28, CDAI, SDAI, HAQ). The final aim is to prove the advantages of volar US evaluation in RA patients.

**Methods:**

42 RA patients have been clinically evaluated for pain and swelling of their hand joints, completed VAS and HAQ questionnaires and underwent both volar and dorsal sonography of the hands during the same day. The US examiner was blinded to clinical assessments and lab results. For each patient 20 joints were assessed by sonography (radiocarpal, intercarpal, metacarpophalangeal (MCP) 2-5, proximal interphalangeal (PIP) 2-5). Carpal joints were only evaluated from dorsal view, while MCPs and PIPs were evaluated both from dorsal and volar aspect resulting a total of 36 distinct evaluations for each patient. GSUS synovial hypertrophy was assessed both by quantitative measurement and semiquantitative scale (0-3 grades); Doppler signal (PDUS) was recorded on a semiquantitative scale (0-3 grades). The semiquantitative grades for both GSUS and PDUS evaluation of each joint were added and the sum was defined as the Echographic Score (ES) of each patient. Separately, we added the semiquantitative grades for volar and dorsal side, resulting in Volar ES (VES) and Dorsal ES (DES) of each patient.

**Results:**

We found ESs correlated with other activity scores: DAS28, CDAI, SDAI, HAQ. Correlations with clinical indices as CDAI and SDAI were stronger for VES than for DES. US discovered more synovitis than clinical examination.

**Conclusion:**

VES is a suitable reflection of RA activity and volar US examination should accompany the dorsal one both in clinical practice and in clinical trials.

## Background

The role of US in evaluating the small joints of RA patients is still debatable in Rheumatology. It has been heavily studied for its potential as an outcome measure, but its standardization remains a problem. Both clinical practice and clinical trials concerning RA are based upon various composite indices for assessing disease activity and responsiveness to treatment (DAS28, HAQ, CDAI and SDAI). They have proven sensitivity to change, validity and reliability; unfortunately they are mainly based on subjective issues (like patient's appreciation of pain). US was proven better than clinical examination in detecting synovitis [1] and, given its objective nature, several authors suggested it should be used instead of clinical assessment in patient evaluation [2].

The concepts of intraarticular fluid and synovial hypertrophy in GSUS are now clearly defined [3] and also the standard position of the patient and transducer for performing US [4]. Being an operator-dependent technique, the reliability and reproducibility of GSUS may be improved by selecting the most appropriate joint recess for the examination, another issue that is still under debate [5].

The generally accepted method for synovial hypertrophy quantification is the semiquantitative scale [6,7], with values between 0-3, where 0 = no intraarticular changes, and 1-3 indicating mild, moderate, and large synovial hypertrophy. The development of an US-based global scoring system is one of the tasks of OMERACT group [8]. A number of scoring systems have already been developed [9-13], focusing mainly upon scanning a limited number of joints to reduce the examination time, and upon the sensitivity to change after remissive treatment but there is limited data regarding the value of volar vs. dorsal US examination of the same joint. In a previous pilot study, we found higher correlations between volar synovitis and clinical findings when compared to the dorsal one [14]. The current study has been conducted on a larger cohort, searching for correlations between US synovitis score (volar and dorsal separately) and the other parameters widely accepted as reliable measures of disease activity. The final aim was to identify the best scanning area (volar or dorsal) to be used for global US scoring in RA.

## Methods

42 RA patients (5 male, 37 female) who were admitted to Sf. Maria Hospital starting from October 2008, selected to have at least one painful or swollen joint have been included in this study. All subjects fulfilled ARA criteria for RA [15] and signed the written informed consent for study participation before the enrollment. The patients were informed about the purposes of the study and about detailed study procedures. With the exception of prolonged US examination, all other procedures of this study are common at the admittance of RA patients in Romania. This study was approved by the medical ethical committee of the "Carol Davila" University of Medicine and Pharmacy Bucharest, Romania. Patients with major hand deformities were excluded from the group, because US images could be altered from the misalignment of hand bones. Mean (SD) age in our group was 55.7 (12.2) years, range 30-83; mean (SD) disease duration was 64.6 (77) months, range 1-300. The patients were included regardless of the treatment they were on at the enrollment. All patients completed Visual Analogic Scale (VAS) evaluation for their pain (VASP), for global activity of their disease (VAS global), and HAQ (Health Assessment Questionnaires). VAS MD was recorded as the clinician's opinion regarding global RA activity of the patients. A clinician, trained in RA assessments, recorded for each patient the number of tender joints (TJC) and swollen joints (SJC). CDAI (clinical disease assessment index) was calculated based upon TJC and SJC, VAS global patient and VAS MD; DAS28 and SDAI (Simplified Disease Activity Index) were then calculated based upon lab results for ESR and CRP [16,17].

The main demographic characteristics of our patients together with clinical and laboratory data in our group are in Table 1.

**Table 1:** Demographic, clinical and laboratory findings in our patients N = 42

	Range	Mean (SD)
age	30.00 - 83.00	55.73 (12.23)

Disease duration (months)	3.00 - 300	64.64 (77.05)

DAS 28	3.46 - 8.30	6.15 (1.18)

VASP	4.00 -10.00	7.93 (1.72)

VAS global	4.00 -10.00	7.67 (1.64)

SJC	1.00 -18.00	7.61 (4.16)

TJC	.00 - 24.00	13.04 (6.31)

VAS MD	2.00 - 93.00	10.69 (17.82)

HAQ	.10 - 2.70	1.55 (.75)

ESR	4.00 - 130.00	50.02 (29.31)

CRP	.00 - 209.30	30.65 (41.39)

CDAI	14.00 - 56.50	33.90 (11.13)

SDAI	15.50 - 264.30	64.23 (46.84)

VASP, Visual Analogic Scale for the patient's pain; VAS global, patient's global evaluation of their disease activity; VAS MD, physician's global evaluation regarding patient's disease activity; SJC, the number of swollen joints; TJC, the number of tender joints; HAQ, Health assessment Questionnaire; ESR, erithrocite sedimentation reaction, CRP, C reactive protein; CDAI, clinical disease activity index; SDAI, simplified disease activity index

### Clinical examination

Clinical examination was performed for all patients by the same physician trained in joints assessment - the examination included all 28 joints from DAS28 [18]. TJC and SJC were recorded as follows: 1 if present and 0 if absent. Consequently, the same physician collected the other variables: the patients' VAS for global activity and pain (0-10), HAQ filled by patients and made his own appreciation of global disease activity on a VAS MD questionnaire. ESR and CRP for all patients were recorded at enrollment time.

### Ultrasonography

US examination was performed later the same day, by a sonographer trained in Musculoskeletal US for 8 years. The machine used for the study was an ESAOTE MyLab 25, with a multifrequency linear 10-18 MHz transducer. The scanning technique and the settings of the machine were the same for all the patients and all examinations were performed in a dark room by the same physician, who was blinded to clinical evaluations. Ultrasonography was performed on 10 joints at both hands, 8 of them in both volar and dorsal aspect (MCPs 2-5, PIPs 2-5). Carpal joints (radiocarpal and intercarpal) were only examined from the dorsal side, because of the special position of carpal bones (we found no data regarding volar incidence for carpal joints in the literature). MCP1 and PIP1 were excluded based upon the rarity of synovitis in these locations in RA. For carpal joints, scanning was performed in a longitudinal plane, from dorsal side, over the surface of radius, lunate and capitate bone [19]. For MCPs and PIPs, scanning was performed longitudinally, over the joint space, first from dorsal and then from volar side. No compression on the transducer was applied. For Doppler signal evaluation, standard Doppler settings of the machine were established: Pulse Repetition Frequency (PRF) was adjusted to maximize sensitivity - from 500 to 750 Hz, the highest gain and high colour persistence without background noise, low wall filter [12,20].

We measured the hypoechoic area between tendons (extensors or flexors) and cortical bone, without differentiating fluid and synovial hypertrophy. This hypoechoic area inside the joint was defined before in literature as synovitis [21]. We performed dorsal measurements perpendicularly to the bone, in the point of the greatest thickness of hypoechoic area as follows: for radiocarpal joint, on top of lunate bone; for intercarpal joint, on top of capitate bone; for MCPs, at the level of metacarpal neck, for PIPs, at the level of the first phalanx [19].

GSUS synovial hypertrophy was assessed both by quantitative measurement and semiquantitative scale (0-3 grades); PDUS was recorded on a semiquantitative scale (0-3 grades). The semiquantitative grades for each joint were added and the sum was defined as the Echographic Score (ES) of each patient. Separately, we added the semiquantitative grades for volar and dorsal side, resulting in Volar ES (VES) and Dorsal ES (DES) of each patient.

A dimension of 0.5 mm was considered the cutoff limit for positive synovitis, and the number of joints with synovitis above this value in each patient was defined as Echographically Positive Joints (EPJ). As a value > 3 mm in hand joints is perceived as large synovitis [6], we counted it as 3 on semiquantitative scale; for an accurate differentiation between grades 1 and 2 we made the transformations as follows: grade1 = synovitis between 0.5-2 mm, grade 2 = synovitis between 2-3 mm. We used both scales of quantification because we only found limited data in literature regarding semiquantitative scale on the volar side. For volar synovitis, we measured the hypoechoic tissue between flexor tendon and cortical bone, perpendicularly to the bone, at the point of its greatest thickness, and we quantified it the same way as the dorsal one. Doppler signal was semiquantitatively quantified, as described in the literature [22-24] on a 0-3 scale (0 = absence, 1 = mild, single vessel signal, 2 = moderate, confluent vessels, 3 = marked vessel signals in more than half of intraarticular area).

### Statistical analysis

We used SPSS 16.0 package; for quantitative parameters we used mean, standard deviation and range. Correlations between different variables were evaluated by 2-sided exact Pearson's correlation coefficients. Any value of p < 0.05 was considered significant.

## Results

We calculated global ES and separately VES respectively DES (see Table 2). DES (mean, SD: 17.78, 6.71) was found higher (t = 17.7, p < 0.01) than VES (11.69, 8,05).

**Table 2:** US parameters in our patients N = 42

	Range	Mean (SD)
SCORECO (ES)	7.00 - 64.00	29.47 (13.50)

VOLAR ES	.00 - 30.00	11.69 (8.05)

DORSAL ES	6.00 - 34.00	17.78 (6.71)

EPJ	2.00 - 31.00	15.33 (7.76)

VOLAR EPJ	.00 - 15.00	6.92 (4.21)

DORSAL EPJ	2.00 - 16.00	8.33 (4.20)

SCORECO, Echographic Score; EPJ, Number of Echographically Positive joints

We examined 16 finger joints (volar and dorsal, as previously described) for each of the 42 patients included in the study (a total of 672 joints for each side). Radiocarpal and intercarpal joints have been assessed from dorsal view only. The highest prevalence of positive synovitis (>1 semiquantitatively) has been found in carpal joints (91%). For the rest of the joints, we found a variable prevalence of positive synovitis from the highest (88.1%) in MCP2 volar side to the lowest (35,7%) in PIP5 volar side.

**Figure 1:**
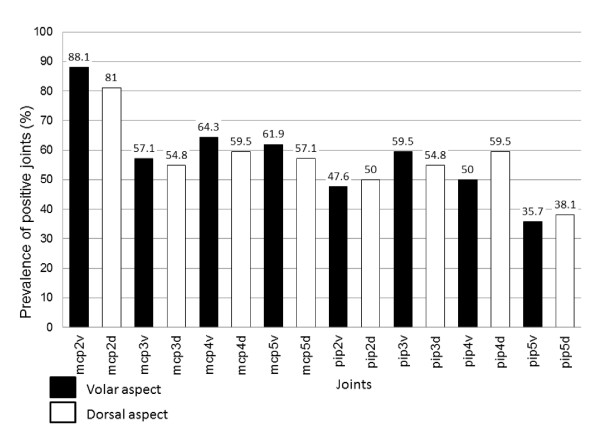
**Prevalence of GSUS synovitis in volar versus dorsal aspect**.

We also counted for each side the number of EPJ (joints that show at least grade 1 synovial hypertrophy at US examination).We discovered a systematic higher prevalence of GSUS positivity on the volar vs. dorsal side (see Figure 1).

**Figure 2:**
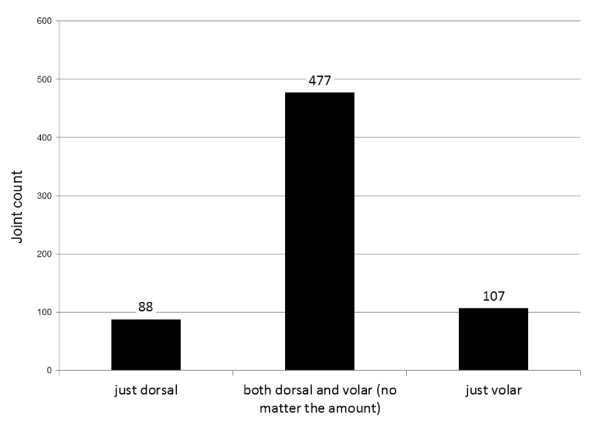
**Qualitative comparison in volar versus dorsal synovitis evaluation**.

In the qualitative analysis, from the total number of 672 joints we found volar synovitis alone (regardless its dimensions on semiquantitative scale) in 107 joints, dorsal alone in 88 joints and 477 joints were echographically identical (positive or negative) on volar and dorsal side (see Figure 2).

In the quantitative analysis, when we compared the dimensions of synovitis (on a 0 to 3 semiquantitative scale), we found higher values for volar than dorsal synovitis in 162 joints, higher dorsal than volar in 128 joints, and in 382 joints synovitis score was identical (p < 0.05). The total number of joints with grade 3 synovitis (the highest size) was found greater on volar side (see Table 3).

**Table 3:** Differences between volar and dorsal aspect on semiquantitative scale (0-3) for synovial hypertrophy

Synovial hypertrophy size	Difference Volar - Dorsal (scale 0-3)	No. of joints
Greater in volar	+3	6

	+2	51

	+1	105

Simmilar in both incidences	0	383

	-1	88

	-2	37

Greater in dorsal	-3	2

**Figure 3:**
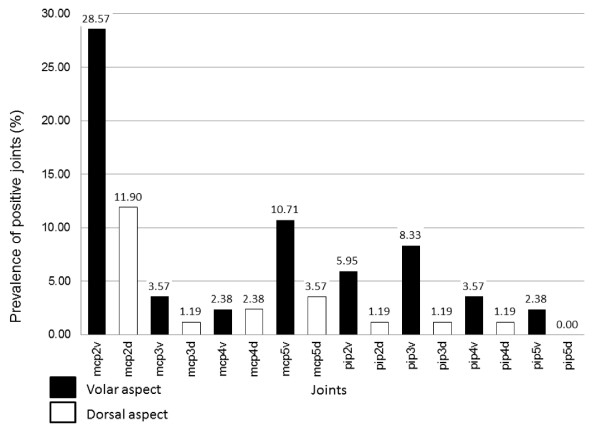
**Prevalence of Doppler positive joints- volar versus dorsal**.

Regarding PDUS, a variable prevalence in our patients was found. Except for MCP2, with a mean Doppler positivity prevalence of 20.23% (28.57% volar and 11.9% dorsal), the mean prevalence for all other joints regarding Doppler signal positivity was very low: 3.57% (5.27% volar and 1.53% dorsal). The PD score resulted significantly higher in volar than in dorsal aspect of joints (t = 4.8, p < 0,001)- see Figure 3.

ES significantly correlated with CDAI (Pearson's index r = 0,628, p < 0,001) and SDAI (r = 0,403, p < 0,01). VES correlated with CDAI (r = 0,470, p < 0.01) and SDAI (r = 0.468, p < 0.01), and DES correlated with CDAI but not with SDAI (r = 0.690, p < 0.01; r = 0,274, p = 0,08). ES also correlated with DAS28 both on dorsal and volar sides (r = 0,407, p < 0,01). ES correlated with HAQ (p < 0.05), on both volar and dorsal sides. We did not find any correlation between ESs and VAS for pain and global activity, whilst a correlation between ES on both sides and VAS MD was detected (p < 0.05).

The number of Echographically Positive Joints (EPJ) significantly correlated with DAS28, TJC, SJC, CDAI, SDAI and HAQ (for all of these p < 0,05). ES and both VES and DES correlated well with TJC and SJC (Tables 4 and 5).

**Table 4:** Correlations between ESs and measures of disease activity

		SJC	TJC	HAQ	CDAI	SDAI	ES	VOLAR ES	DOR ES
SJC	Pearson Corr.	1	,339*	,330*	,622**	,533**	,432**	,385*	,406**

	Sig. (2-tailed)		,028	,033	,000	,000	,004	,012	,008

TJC	Pearson Corr.	,339*	1	,641**	,813**	,310*	,430**	,325*	,476**

	Sig. (2-tailed)	,028		,000	,000	,046	,004	,036	,001

HAQ	Pearson Corr.	,330*	,641**	1	,608**	,516**	,321*	,291	,296

	Sig. (2-tailed)	,033	,000		,000	,000	,038	,061	,057

CDAI	Pearson Corr.	,622**	,813**	,608**	1	,574**	,591**	,471**	,625**

	Sig. (2-tailed)	,000	,000	,000		,000	,000	,002	,000

SDAI	Pearson Corr.	,533**	,310*	,516**	,574**	1	,351*	,379*	,251

	Sig. (2-tailed)	,000	,046	,000	,000		,023	,013	,109

ES	Pearson Corr.	,432**	,430**	,321*	,591**	,351*	1	,929**	,897**

	Sig. (2-tailed)	,004	,004	,038	,000	,023		,000	,000

VOLAR ES	Pearson Corr.	,385*	,325*	,291	,471**	,379*	,929**	1	,670**

	Sig. (2-tailed)	,012	,036	,061	,002	,013	,000		,000

DOR ES	Pearson Corr.	,406**	,476**	,296	,625**	,251	,897**	,670**	1

	Sig. (2-tailed)	,008	,001	,057	,000	,109	,000	,000	

**Table 5:** Correlations between ESs, no of EPJ and other measures of disease activity

		das28	VAS MD	ES	VOLAR ES	DOR ES	EPJ	VOLAR EPJ	DORSAL EPJ
das28	Pearson Corr.	1	,342*	,394**	,374*	,345*	,390*	,373*	,341*

	Sig. (2-tailed)		,027	,010	,015	,025	,011	,015	,027

VAS MD	Pearson Corr.	,342*	1	,245	,289	,146	,293	,265	,278

	Sig. (2-tailed)	,027		,118	,064	,357	,060	,090	,075

ES	Pearson Corr.	,394**	,245	1	,929**	,897**	,935**	,868**	,848**

	Sig. (2-tailed)	,010	,118		,000	,000	,000	,000	,000

VOLAR ES	Pearson Corr.	,374*	,289	,929**	1	,670**	,851**	,917**	,653**

	Sig. (2-tailed)	,015	,064	,000		,000	,000	,000	,000

DOR ES	Pearson Corr.	,345*	,146	,897**	,670**	1	,859**	,647**	,923**

	Sig. (2-tailed)	,025	,357	,000	,000		,000	,000	,000

EPJ	Pearson Corr.	,390*	,293	,935**	,851**	,859**	1	,904**	,921**

	Sig. (2-tailed)	,011	,060	,000	,000	,000		,000	,000

VOLAR EPJ	Pearson Corr.	,373*	,265	,868**	,917**	,647**	,904**	1	,672**

	Sig. (2-tailed)	,015	,090	,000	,000	,000	,000		,000

DORSAL EPJ	Pearson Corr.	,341*	,278	,848**	,653**	,923**	,921**	,672**	1

	Sig. (2-tailed)	,027	,075	,000	,000	,000	,000	,000	

## Discussion

Our data show the advantages of the volar US examination over the dorsal one in RA synovitis assessment - its sensitivity qualifies for becoming a global outcome measure. Apart from that, the present study evaluated GSUS and PDUS additive scores (ESs) in parallel with physical examination, subjective evaluation of disease pain and global activity and traditional tools for disease activity evaluation in RA. US data have a higher significance when they are accurately compared to other measures of disease activity, in particular because they need to be measured regularly at 3 months interval, in order to better control the disease [25]. In fact, we felt the need to establish as accurately as possible a more objective measure based not only on clinician's feeling, but also on their ability to correctly depict and quantify synovitis. The role of US is to complement clinical examination that sometimes can lead to false positive or false negative results [22]. As mentioned before, OMERACT is in preparation for a global US score [8], combining best parts of all scores described so far, in order to reach maximal reliability, validity and responsiveness, integrated together as OMERACT filter [26]. It is, though, of highest importance to establish which joint recess (dorsal or volar) is best correlated with accepted clinical tools for RA activity quantification [5].

Our first pilot study on this matter (2005) stated that volar synovitis is more correlated to clinical examination than dorsal one [14]. Several studies have addressed volar synovitis so far [11,27,28]. In 2004, Hoving et al stated that in hand joints small amount of fluid is best visualized from volar side with fingers in gentle flexion [27]. In a recent scoring system, Backhaus found volar synovitis present in 86% of affected joints, whilst dorsal synovitis alone in only 14% [11]. Ostergaard and Szudlarek found only 33% of patients having synovitis on both volar and dorsal side; in the majority of their cases synovitis was limited to volar- 43% or dorsal side - 27% [28]. In our study, we did both qualitative and quantitative synovitis assessments - in the qualitative analysis we found echographically identical synovitis (positive or negative) on volar and dorsal side in 477 joints, with volar alone positivity higher. In the quantitative analysis, when we compared the dimensions of synovitis (0-3 semiquantitative scale), we found higher values for volar than dorsal synovitis in 162 joints, higher dorsal than volar in 128 joints, and in 382 joints synovitis score was identical (p < 0.05). No other statistical correlations between the two sides, joint by joint, were found in the literature.

According to literature, volar synovitis is always found on the proximal area of MCP and PIP joints [28]. We used the same method previously described [28] for depicting volar synovitis: with the transducer placed longitudinally, without compression, on the median line of the joint, measuring the hypoechoic area between flexor tendon and bone contour, proximal to volar plate. As we found no published correlations between quantitative and semiquantitative scale for volar synovitis, we used our own scale: 0.5-2 mm = grade 1, 2-3 mm = grade2, >3 mm = grade3 (same correspondence as for dorsal synovitis evaluation).

Our results are consistent with literature data as we found a higher percentage of positivity on GSUS but also on PDUS on the volar side. As the study involves calculating ES as the sum of semiquantitative values of GSUS, we also calculated ES for volar and dorsal side, separately. We found all ESs significantly correlated with standard measures of disease activity (DAS28, CDAI, SDAI, SJC, TJC, HAQ). We found the differences in synovitis depicting between the two aspects of joints statistically significant, especially regarding PDUS (p < 0.001). We found no previous data in literature regarding comparison of PDUS on volar vs dorsal sides.

Data in recent literature emphasize the necessity of establishing "target joints", meaning joints most frequently involved in RA for scoring global synovitis, in order to shorten the time of US examination [11-13]. Wrist being the most affected joint in RA (mean of 67% of cases), it has been selected as a" target joint" in clinical trials, being used in most of the scores available to date. Except for the wrist, different scores used different "target joints": MCPs 2, 3, 5, PIPs 2,3 and MTPs. We found positivity prevalence of MCP4 comparable to MCP3 or 5, probably because of volar synovitis contribution (in most of the previous studies, MCP4 was not considered "target joint"). - see Figure 1

**Figure 4:**
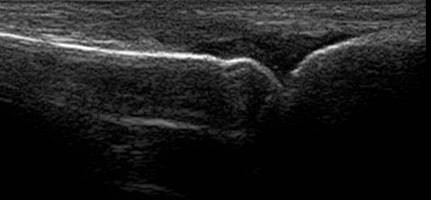
**Dorsal synovitis - MCP 3 joint**.

**Figure 5:**
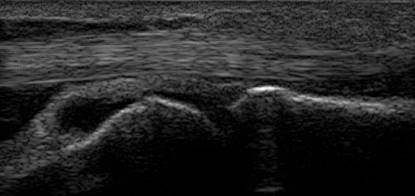
**Volar synovitis - MCP 3 joint**.

**Figure 6:**
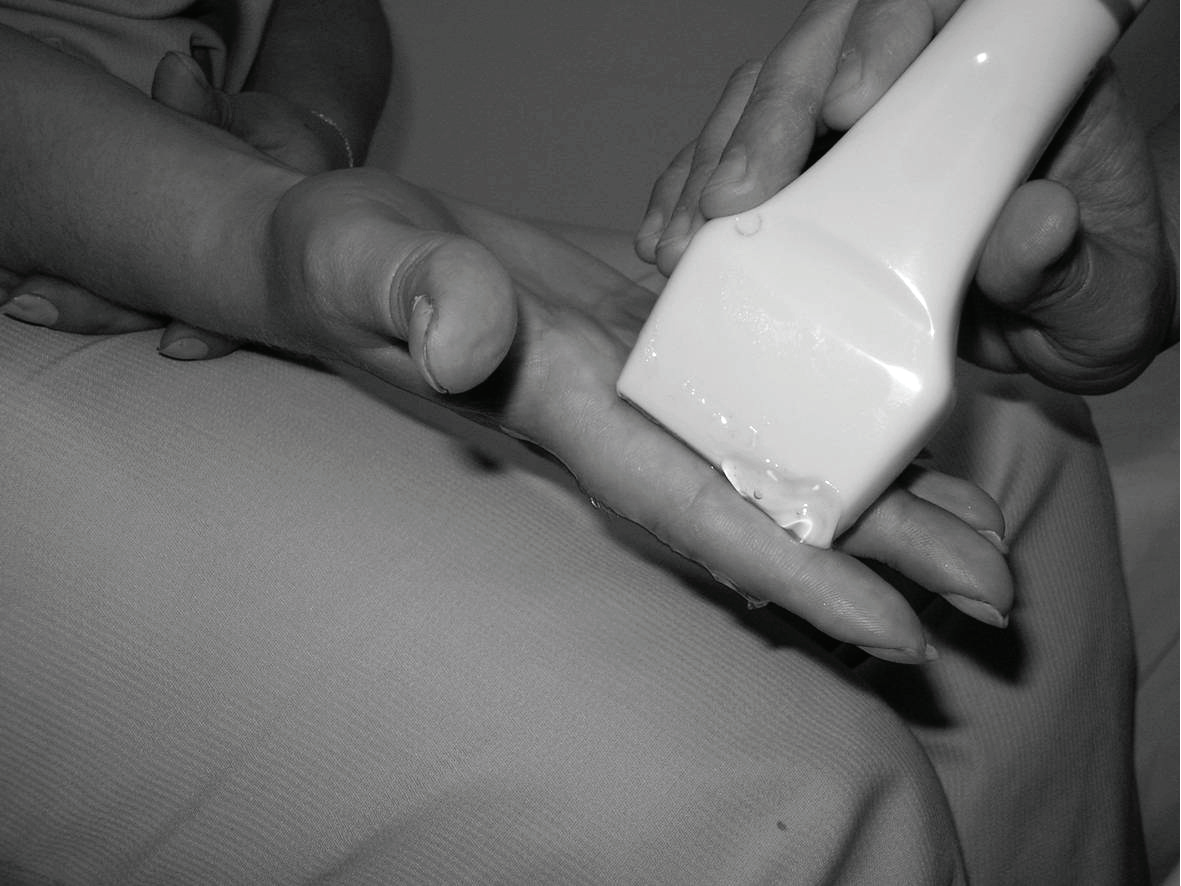
**Transducer position for volar synovitis depicting**.

As a personal observation, we agree with the conclusion of Hoving [27], that volar synovitis is more easily depicted and quantified in small hand joints than the dorsal one, probably due to the flexor tendon position towards the joint- more distant from the joint comparative with extensor tendon, due to the presence of volar plate (images of synovitis in MCP3 in both dorsal and volar aspects in Figures 4,5). The position of the hand and transducer for volar synovitis depicting is represented in Figure 6.

## Conclusion

Volar US examination of the hand depicts more synovitis in joints affected by RA and more PDUS was found on that aspect of joints- it should be used in clinical evaluation and also in clinical trials in addition to the dorsal one, as it was also correlated with all signs of RA activity previously accepted. Scoring both dorsal and volar synovitis could be the best sonographer's option to reflect disease activity in RA. However, the new scores need to be verified regarding their sensitivity to change. Further studies on the volar area- which allows an easier visualization and grading of synovitis for MCPs and PIPs and, though, could prove higher interobserver agreement (repeatability) than the dorsal one in clinical practice and clinical trials- are warranted.

### Ethics approval

This study was approved by the medical ethical committee of the "Carol Davila" University of Medicine and Pharmacy Bucharest, Romania.

## Competing interests

The authors declare that they have no competing interests.

## Authors' contributions

All authors were involved in drafting this article and they all approved its final version for publication. Study design and concept: VV, DP, RI, FB Literature research: AB, VB, CC US examinations: VV Clinical examinations: SL Acquisition of data: AB, VB, CC, MA Statistical analysis: FB Analysis and interpretation of the data: FB, VV, RI.

## Pre-publication history

The pre-publication history for this paper can be accessed here:

http://www.biomedcentral.com/1471-2474/12/124/prepub
